# A new endoscopic submucosal resection –ligation technique for gastric tumors 

**Published:** 2020

**Authors:** Hamid Asadzadeh Aghdaei, Amir Sadeghi, Ahmad Ghorbanpour Nouri, Gholam Reza Nouri, Arash Dooghaie Moghadam, Mohammad Reza Azizi, Pegah Eslami

**Affiliations:** 1 *Basic and Molecular Epidemiology of Gastrointestinal Disorders Research Center, Shahid Beheshti University of Medical Sciences, Tehran, Iran*; 2 *Gastroenterology and Liver Diseases Research Center, Research Institute for Gastroenterology and Liver Diseases, Shahid Beheshti University of Medical Sciences, Tehran, Iran *

**Keywords:** Subepithelial Tumor, Endoscopic Mucosal Resection

## Abstract

Although most subepithelial lesions are benign, the malignant forms could present as serious life-threating cancers. Their accurate diagnosis depends on complete surgical resection. Different endoscopic methods have been recommended for the resection. Recently, the EMR has been considered as a safe and effective technique, while various revised EMR techniques have been introduced. In this study, a new version of EMR has been evaluated in two patients. Two middle-aged cases with gastric subepithelial tumors were admitted to Taleghani gastrointestinal department. The polyps were resected via our new Endoscopic Mucosal Resection (EMR) technique. In this technique, the needle knife was used for un-roofing the mucosal surface. Then, the polyps were resected with hot snares. The hemoclips were applied for ligation too. We found no early or delayed complications. Furthermore, the microscopic margins of the lesions were free. Our study represented a safe and cost-beneficial technique for subepithelial lesions and no complications was found and the margins were free. However, further investigations are required for confirming the validity of this new EMR technique.

## Introduction

 Subepithelial tumors (SETs) include a wide range of spectrum from benign to malignant forms of polyps. These lesions can be either serious such as GIST or benign such as lipoma, leiomyoma, or schwannoma (1). Retrospective studies have revealed a prevalence of 0.36% for SETs on a routine endoscopy (2). In addition, the incidence rate of malignancy is about 3% in subepithelial lesions (3). Unfortunately, stacked biopsies cannot provide sufficient histologic specimens for certain diagnoses. Accordingly, complete resection is necessary for evaluating the type of lesion (8). Although surgical resection has been considered as the routine method for subepithelial tumor resection, non-invasive options are increasingly used in polyp resection (4). Recently, in several situations, endoscopic resection of subepithelial lesions as a less invasive option has replaced surgical resection (5). Different endoscopic methods with minimum side effects have been introduced with the purpose of full-thickness resection (2). In this study, we will present two cases to introduce a new version of Endoscopic Mucosal Resection (EMR). Herein, through these two cases, we explain the safety, efficacy, and clinical outcome of our new version of EMR-L technique. 

## Case Presentation

***Case presentation 1***

An Iranian 50-year-old man attended Taleghani Gastrointestinal Clinic for recurring epigastric pain. This patients complaints included epigastric pain, heartburn and dysphagia. He suffered from these signs and symptoms from four months before the tumor detection. No weight loss was detected. Past surgical history and family history of the patient were negative too. He did not smoke or drink alcohol. He was treated several times with omeprazole, anti-acids, and baclofen. Finally, he was referred to Taleghani Hospital Clinic by food sensation. In our setting, he was evaluated by endoscopy and a submucosal mass in cardia was detected.

On physical examination, his temperature was 37.1°C, heart rate was 82 beats per minute, respiratory rate was 10 breaths per minute, and blood pressure was 130/80 mm-Hg. Abdominal examination showed neither distention nor tenderness. No organomegaly or palpable mass was found. Furthermore, laboratory tests had no remarkable findings. The evaluations included endoscopy of upper and lower gastrointestinal tract at the same time. The result of colonoscopy was normal. Endoscopy of the upper GI tract showed a 19×13 mm homogenous and hypoechoic submucosal lesion, with smooth and defined border in cardia at the third layer. In addition, EUS reported a small calcification in the center of lesion.

***Case presentation 2***

An Iranian 40-year-old lady was referred to the gastroenterology department of Taleghani Hospital with a submucosal mass lesion. The patient described her symptoms as intermittent epigastric pain, discomfort, dyspepsia, and heartburn from more than six months before the admission. No significant weight loss was detected in our case. She revealed two caesarean sections as the past medical history and the family history of gastrointestinal malignant disorders was negative. In addition, she denied any positive history of alcohol drinking or smoking.

On physical examination, her temperature was 37.6°C, heart rate was about 84 beat per minute, and blood pressure was 110/70 mm-Hg. Abdominal examination showed a soft and non-tender abdomen with no sign of organomegally or palpable mass. The rest of the physical examinations and laboratory tests were normal.

The evaluations before the referral included upper and lower GI tract endoscopy and ultrasonography. Endoscopy reported a polyp-like lesion in the antrum, while the mucosa in this place was normal. In addition, EUS showed a 16.3 mm subepithelial, iso-hyperechoic lesion originating from the submucosal layer. The pillow sign of the mass was negative. The gross feature of the lesion was in favor of lipoma.

***Endoscopic devices and procedures***

A soft, straight, transport cap with an inside rim (D-201-11802, Olympus) was fitted on to the tip of a standard single channel endoscope (GIF-260, Olympus). Other devices included Needle Knife (Olympus), Hot snares, and Hemoclips (Micro-Tech Nanjing Co., Ltd.).

**Figure 1:**
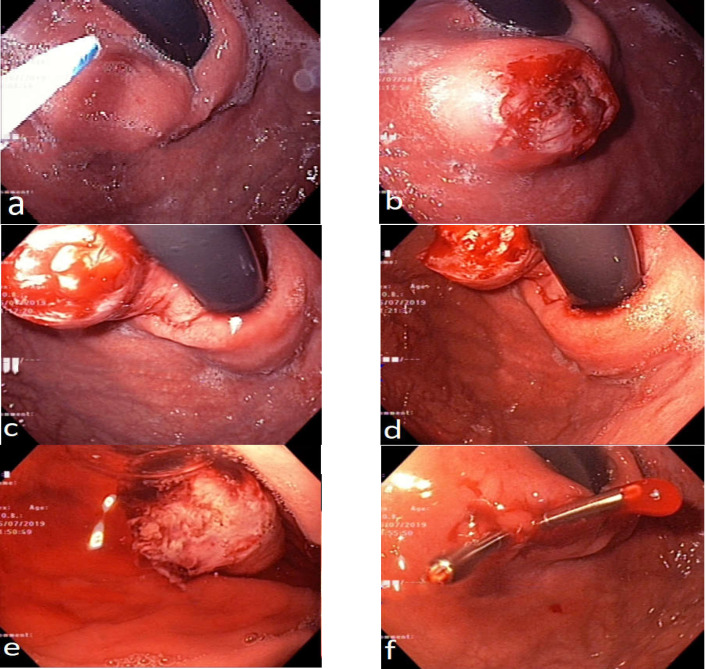
Subepithelial tumor resection with endoscopic mucosal resection technique using hemoclips for ligation. **a) **The submucosal lesion was detected by retroflexed maneuver. **b) **The mucosal surface was unroofed with needle knife. **c, d)** The mass was enucleated. **e) **It was resected with a standard polypectomy hot snares. **f) **Two hemoclips were inserted on the base of the lesion

Under sedation with intravenous propofol in standard position, endoscopy was performed. The procedures included the following steps and all of them were attended by an expert and single endoscopist (Figure 1). 

 The submucosal lesion was detected by retroflexed maneuver. Then, after the estimation the site of the polyps. The mucosal surface was unroofed with needle knife (this part is different with other previous known methods.). The lesion was retracted with grasps, and the mass was enucleated. It was resected with standard polypectomy hot snares. After resection, the mass was sent to a pathologist. Two hemoclips for each mass were inserted on the base of the lesion (Figure 1). ***Follow-up assessment***

In both cases, second look endoscopy was performed after six hours of the resection. The outcomes were followed up by endoscopy after the two months following the procedure, to confirm the healing of the artificial ulcers and other complications. The assessment of endoscopic parameters included resection rate, early and delayed perforation rate, early and delayed bleeding rate, dehiscence rate, and recurrence rate.

***Histopathological examinations***

In both cases, the tissue specimens were fixed in formalin solution. The histological analysis included the identification of cell types, nuclear atypia, histopathological type, tumor size, and tumor invasion. In the first case, immunohistochemical study was also performed. Furthermore, the margins of resection were examined macroscopically and microscopically.

***Histopathological results***

***Case 1***

The specimen consisted of a polypoid creamy tissue fragment measuring 1.7×1.5×1 cm and tiny fragments of creamy soft tissue measuring 1×0.7×0.3 cm. \4The primary histopathological analysis suggested gastrointestinal stromal tumor (GIST). IHC study included Smooth Muscle Antibody (SMA), Desmin, CD117, and DOG1. Desmin and SMA were positive but CD117 and DOG1 were negative. IHC and histologic findings were in in line with leiomyoma.

***Case 2***

The specimen consisted of adipose tissue fragment measuring 1×1×0.5 cm. Histologic findings of endoscopic biopsy confirmed lipoma.

***Follow up***

Re-endoscopy, after six hours of resection reported no early complication, in both cases. After 12 hours of the procedures, both patients were discharged. Once discharged, the patients were prescribed daily oral Proton pump inhibitors (PPI) for two months. After two months, patients were evaluated by endoscopy again. Repeated endoscopy revealed, the hemoclips were spontaneously removed and the mucosa of the antrum was normal, in both cases. No macroscopic evidence of tumor was detected. Furthermore, the resection margins of the polyp were evaluated microscopically. Microscopically, all margins were free. Delayed perforation or delayed bleeding were not detected after 2 months.

## Discussion

Gastric SETs are usually detected incidentally during the upper GI tract endoscopy (6). Although SETs are usually benign, some of them could be malignant. One of the malignant types of SETs is GIST, which must be completely resected (7). Unfortunately, simple biopsy cannot lead to accurate diagnosis of SETs (8). For the same reason, several techniques have been recently introduced for the diagnosis of subepithelial lesions (3). EUS evaluation is a helpful method for identifying the wall layer in lesions, as well as for accurately measuring the tumor size, cystic components, and irregular margins (6, 9). The common sampling techniques include EUS-FNA, core biopsy under the guide of EUS, single incision needle knife, and endoscopic submucosal resection techniques (3). However, EUS-FNA and core biopsy have more serious complications, including penetration, in comparison to Endoscopic Mucosal Resection (10). On the other hand, immunohistochemical staining is an essential requirement in diagnosis of subepithelial lesions, which needs as many tissues as possible. Meanwhile, these two methods provide limited tissue samples. Resection of submucosal lesions with an endoscope has been reported using a variety of techniques, ranging from simple snare resection to endoscopic submucosal dissection. However, the result has been variable with respect to complete resection and complications (2). Endoscopic Mucosal Resection (EMR) compared to Endoscopic Submucosal Dissection (ESD) is considered a conservative method with less complications and less waste of time. Further, this method is less technique dependent. However, it seems ESD is more effective in deep and large tumors and less recurrence has been detected via this method in comparison to EMR (11). Thus, recent studies have focused on determining new versions of EMR to improve its efficacy which could lead replace to ESD (12). The new versions of EMR, including EMR with ligation (EMR-L), EMR with double ligation (EMR_DL), and EMR after circumferential pre-cutting (EMR-P) could help in deeper resection with less positive basal margins and complications (12). EMR-L is regarded as a less invasive technique, suitable for tumors with low risk of invasion (13). In comparison to surgical resection techniques, unfortunately, EMR-L increases the risk of incomplete resection (14). Cap endoscopic mucosal resection (EMR-LC) is a new type of EMR technique. In this method, the incision with knife at the first step is not obtained and the resection afforded via electrosurgery and the ligation was used for enucleation (12). A recent study compared the ESD technique with LC-EMR in terms of their impact and complications in carcinoid tumors. Although the LC-EMR was reported as an effective technique, all of the lesions were less than 10 mm. The impact of this method on large size polyps has been unclear (12). EMR-P is a common technic for subepithelial lesions resection. In this method, a circumferential lesion around the surface of lesion is performed and a snare is used for dissection. On the other hand, ESD is similar to EMR-P, but instead of snare, a tipped knife is recruited (15). In this revised form of EMR techniques, we cut the mucosa with a needle knife. Thereafter, we enucleated and resected the mass with a standard polypectomy snare. Finally, the site of the wound was closed with two hemoclips. However, we did not employ the ligation. Our technique appears to be effective in large size tumors. Furthermore, unroofing the lesion before the incision makes the mass visible and facilitates the removal procedure. Ligation with hemoclips is also a good method for elderly patients with arrhythmia and pacing, in comparison to band ligation. It seems our method had shorter healing rehabilitation period, in comparison with previous methods. Further, we detected no complications after using this method. It seems use of snare and needle knife could reduce the perforation and bleeding rate. In addition, previous studies reported more frequent involved margins via the EMR techniques in comparison to ESD techniques or surgical investigations. This technique as a cost benefit resection method helps treat patients with submucosal lesions by standard endoscopy without hospitalization. However, more investigations with a large study population is required to estimate the efficacy of our technique in diagnosis and treatment of polyps with various sizes.
